# The costs of inappropriate referral pathways in inpatient care for three major noncommunicable diseases in Mongolia: a national registry-based analysis

**DOI:** 10.1186/s12913-021-07281-8

**Published:** 2021-11-27

**Authors:** Ariuntuya Tuvdendorj, Otgonjargal Dechinkhorloo, Bayarsaikhan Dorjsuren, Erik Buskens, Talitha Feenstra

**Affiliations:** 1 Department of Health Policy, School of Public Health, Mongolian National University of Medical Sciences, Zorig street, Ulaanbaatar, 14210 Mongolia; 2 Department of Epidemiology, Groningen University, University Medical Center Groningen, Groningen, the Netherlands; 3 General Authority for Health Insurance, Ulaanbaatar, Mongolia; 4 Department of Health Systems Governance and Financing, World Health Organization, Geneva, Switzerland; 5 Groningen University, Groningen Research Institute of Pharmacy, Groningen, the Netherlands; 6 Centre for Nutrition, Prevention and Health Services, Institute for Public Health and the Environment, Bilthoven, the Netherlands

## Abstract

**Background:**

Non-communicable diseases (NCDs) consistently pose a huge economic burden to health systems and countries in general. The aim of this study was to quantify inpatient costs associated with chronic obstructive pulmonary disease, stroke and ischemic heart disease stratified by type of referral pathway, and to investigate key factors that drive these costs.

**Methods:**

A registry-based data analysis was performed using national public hospital inpatient records from 2016 to 2018 for 117,600 unique patients and linking patient-level inpatient health care use with hospital-specific unit cost per bed-day. These were combined to calculate the annual inpatient costs for each of the three disorders per person and per year. Generalized linear modeling was used to assess the association of inpatient costs with age, gender, location, comorbidity, treatment referral pathways and years.

**Results:**

Across three diagnoses, the majority of patients were female. Most were over 50–60 years old, with more than half being a pensioner, typically with at least one comorbidity. About 25% of patients followed what might be considered inappropriate (unofficial) inpatient referral pathways. Mean annual inpatient costs were int$ 721. These costs rose to int$ 849 for unofficial pathways and dropped to int$677 for official pathways. Further covariates significantly associated with high inpatient costs were location, age, gender, and comorbidity.

**Conclusion:**

Our findings provide background information essential to develop evidence-based and cost-effective interventions aimed at health promotion, prevention and service delivery. Reducing the unofficial use of inpatient care can improve efficient resource allocation in health care and prevent further escalation of inpatient costs in the future.

**Supplementary Information:**

The online version contains supplementary material available at 10.1186/s12913-021-07281-8.

## Key points

- Mean inpatient costs associated with chronic obstructive pulmonary disease, stroke and ischemic heart disease were significantly higher for unofficial referral pathways compared to official referral pathways.
- Key drivers of high inpatient costs next to referral pathway were location, age, gender and comorbidity.
- Factual costs of hospital care for the NCDs considered provide insight in healthcare delivery, and the financial burden of disease for Mongolia and comparable developing countries.

## Introduction

The costs associated with non-communicable diseases (NCDs) consistently pose a high economic burden on health systems and countries in general [1, 2]. With an increasing prevalence of NCDs, the main challenges for health system financing in many low- and middle-income countries (LMICs) are ensuring financial sustainability and efficient resource allocation in health care [3]. Globally, average per capita health spending is approximately US$ 1100, but in absolute terms most LMICs spend less than US$ 130 [4]. The health system financing in LMICs needs to support efficient resource allocation with a particular focus on controlling costs associated with NCDs. This implies reducing inefficient referral pathways. Additionally, prevention policy could help to reduce the need for hospitalizations. Accurate information on the costs related to hospitalizations for NCDs and the factors that influence these costs may inform local policy makers on the provision of service delivery and support prevention and control of NCDs.

One of the factors might be hospital referral pathways. Lack, either real or perceived, of quality access to or the capacity of community level hospitals may lead to inefficient treatment pathways, with direct entry into secondary or tertiary levels of care. Higher level facilities are often better equipped and employ more specialized personnel, hence self-referral tends to occur [5]. However, tertiary hospitals also quickly become overcrowded and health systems endeavor to transfer patients to lower-level hospitals [6–8].

In response, the length of stay for stroke patients has been reduced to about 6 days, while some hospitals transfer patients to lower-level hospitals [9]. Previous evidence suggests that about 20–40% of resources spent on health care may be wasted due to inefficiency and unofficial service use [10]. Inefficient referral may cause unofficial service use. Hence it is relevant to specifically investigate the impact of referral pathways on treatment costs, especially for LMICs.

Mongolia is a LMIC with a population of 3.3 million [11, 12]. The country’s health infrastructure is challenged by the extremely low population density over a large territory under rapid economic transition. The health system is largely hospital-based, with about two third of health financing being spent to inpatient care, and the remaining one third for outpatient care and medicines [5]. This practice pathway emerged during the era of free access to publicly-funded hospitals [13]. Currently, NCD related inpatient care is included in the social health insurance benefits package. Service providers are paid according to a diagnosis-related groups (DRG) payment mechanism [14]. About two thirds of public health spending on NCDs in 2013 (56.1 billion Mongolian National Tugrik (MNT) out of 85.2 billion MNT) was for inpatient care (hospital admission) [15]. Despite apparent unique challenges for the provision of service delivery in Mongolia, elsewhere NCDs are similarly the leading cause of death and disability, responsible for almost 80% of all deaths in 2018 [16–18]. In many aspects, Mongolia displays trends very similar to those previously signaled for LMICs in general: a rise in life expectancy (by 5.6 years over the period 20 years), accompanied by an increase of NCDs. The percentage of all deaths that could be related to NCDs were 80% in Mongolia, 82% in Iran, 89% in China and 77% in Vietnam in 2018, with the remaining deaths related to communicable diseases and injuries [19, 20]. Meanwhile, an increasing prevalence of lifestyle related risk factors for NCDs is observed. The need to better understand what drives hospital costs for NCDs in Mongolia, as well as in other LMIC countries is clear.

Previous evidence showed that inappropriate hospital admissions accounted for 21.7% for public hospital admissions in the department of internal medicine, while this was even higher for private hospitals (27.66%) in Mongolia [21]. This study argued that poor gatekeeping capacity at the primary level of care, a persistently inpatient oriented health system and inefficient provider payment mechanism may explain the relatively large number of inappropriate admissions, leading to unnecessary costs in healthcare [13].

Inpatient costs for NCDs and specifically for Stroke, Chronic obstructive pulmonary disease (COPD) and ischemic heart disease (IHD) have been shown to vary with several factors. The most studied factors that contribute to high inpatient costs in LMICs were age, gender, comorbidities, length of stay, and type of health care providers [22–25]. Also, one study investigated the effect of different referral systems, but this was published in Farsi [7]. Disease specific costing studies thus far were often conducted in a single hospital, for instance in China [26] and in Vietnam [27] were limited by small sample size (*n* = 57 participants). Generalizability to countrywide costs may thus be limited for studies performed in LMICs [6, 26]. On the other hand, overview studies provide insight into global estimates of inpatient costs, however these are very general estimates, and usually not based on patient level data [28].

In Mongolia, no study so far addressed disease-specific inpatient cost, and studied key factors that contribute to high inpatient costs. Stroke, COPD, and IHD are 3 NCDs with large burden of disease in Mongolia. Moreover, their future burden maybe expected to rise, when current trends in lifestyle risk factors like overweight and smoking are not bent towards more healthy pathways.

Information on the inpatient costs for NCDs can be used to prioritize prevention policy focusing on risk factors for NCDs. Evidence on key factors that impact on high inpatient costs can help to control the rise of costs due to inefficient service delivery in practice. With lack of disease-specific costing information and key factors influencing these costs, inefficiency in resources allocation may continue to exist. Furthermore, lack of cost estimates may lead to over or under estimated true intervention costs in applied studies and to ignorance of costs drivers.

The current study therefore adds to this literature. Using a large patient-level dataset from the national Mongolian hospital inpatient registry, in combination with data from hospital financial accounts, information beyond what has previously been published may be retrieved [15]. The database comprises information on patient characteristics and their utilization of inpatient care over a three-year period. In this study, inpatient costs were estimated using a health care provider perspective, while distinguishing a range of cost drivers. The aim of the study was to estimate population mean inpatient costs associated with three diagnosis-related groups (DRGs) of diseases between 2016 and 2018, and to investigate key factors that drive high inpatient costs for these 3 DRG groups in Mongolia, in particular the difference in costs between patients using official and unofficial referral pathways.

## Methods

### Study design

A ‘real world data’ registry-based cost analysis was performed using national public hospital inpatient records for the years 2016 through 2018 in Mongolia. Two data sources were combined to calculate the population mean costs associated with hospitalization for patients with stroke, chronic obstructive pulmonary disease (COPD) and ischemic heart disease (IHD). First, micro-level hospital registry data was used to analyze the characteristics of inpatient care use across different diagnosis-related groups (DRGs). Second, hospital-specific funding source data were collected from hospital financial reports to calculate the unit cost per bed-day. Public funding sources, that is, direct government funding and health care insurance for each type of hospital, were tracked over time. Inpatient health care use was linked with cost per bed-day in order to quantify the costs per patient per year (PPPY). This was repeated for each of the three conditions. Generalized linear modeling analysis (GLM) was used to relate the PPPY to several covariates reflecting patient characteristics, type of treatment referral pathways and diagnosis.

### Data sources

The national inpatient records are managed by the Health Development Center, which is the public authority responsible for collecting, inspecting and annual reporting of national health indicators in Mongolia. The registry uses the international classification of diseases version 10 (ICD-10). We selected the three most prevalent DRGs among the NCDs: COPD (J40-J44), IHD (I20-I25), and stroke (I60-I69).

Inpatient record data contains information on demographics (i.e., age, sex, place of residence, and education), socioeconomic characteristics (social status, working area and profession), medical condition (diagnosis, number of bed-days,) and type of hospitals (regional hospital, province hospital, state clinic, soum hospital, inter-soum hospital). An anonymized patient identification number was used to record any comorbidity as present in a given year. Inpatient care use was estimated at the patient-level rather than per admission only, meaning that for a patient who was admitted to the hospital multiple times or to multiple hospitals, the different episodes of inpatient care costs were summed, to total annual costs for this individual.

Laws on transparency in Mongolia, effective as of 2015, all state authorities, including public hospitals, are obliged to report financial information online and make it freely accessible to the public. Hospital financial reports contain information on total funding and funding sources. The extracted data were further validated through expert consultations.

### Participants

**Fig. 1:**
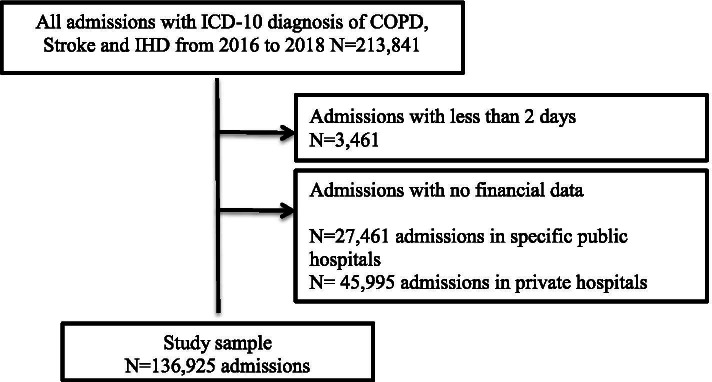
Selection criteria for study sample

Our dataset consisted of all inpatient admissions in Mongolia in the years 2016 to 2018 (Fig. 1). Admissions that had less than 2 bed-days were excluded, to avoid inappropriate inclusion of day-care services and regular outpatient visits. Also, due to unavailable financial information, admissions in private and specific public hospitals that provide inpatient care for specific groups including the railway, court and military sector workers rather than general population were excluded.

A total of 136,925 admission records were selected from 303 public hospitals, including 228 community (soum) hospitals, 39 inter-community hospitals (all primary level care), 16 provincial (aimag) general hospitals, 5 regional diagnostic centers, 12 district general hospitals in Ulaanbaatar (all secondary level care), and 3 general clinic hospitals (tertiary level care).

**Fig. 2:**
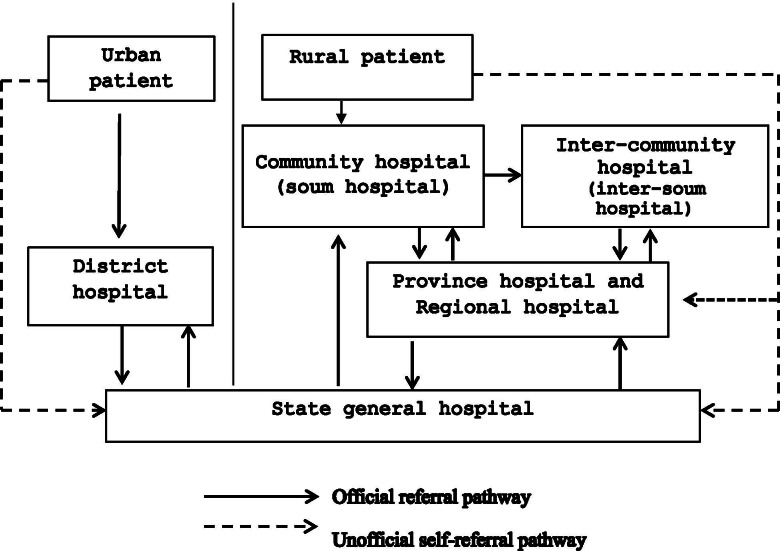
Referral pathway

Patients living in rural and urban areas follow different inpatient care pathways which reflect geographical circumstances (Fig. 2). Generally, patients living in the capital city receive inpatient care from district level hospitals [29]. Their health care provider then decides whether they need referral to a general hospital for specialized care. Similar procedures apply to patients in rural areas. Depending on the proximity of the hospital, patients in rural areas first receive inpatient care at community or inter-community hospitals, and if they cannot be treated effectively, these hospitals refer patients to provincial or regional hospitals, which may, if needed, further refer patients to tertiary level care (the general clinic hospitals in Ulaanbaatar). However, in practice, some patients bypass community and district hospitals and go straight to secondary or tertiary levels care, thus following unofficial referral pathways [5].

### Variables

The outcome variable in the analysis was total hospitalization cost per patient per year. Since price levels varied over time, they were adjusted to the price level of 2018, based on the consumer price index for all 3 years in the final analyses. The primary covariates included in the model were gender, age, comorbidities, location of the patient, social status, treatment referral pathways and year. Sex was dichotomized (female versus male); age was categorized into ten-year age groups: 0–40, 40–50, 50–60, 60–70, 70–80 and 80 and older; patient location was dichotomized (urban versus rural); and patient comorbidities were identified within the three ICD codes recorded during each hospitalization. Social status was categorized into 5 categories: formal sector salaried employee, private sector employee, herders, pensioners and others, including full-time students and children aged under 15 years. Patient referral pathways were assessed and coded as dummy variables based on each individual patient’s admissions. The dummy variable indicated whether the treatment path was an official or unofficial referral by merging information on referral paths with patient location information over the one-year period.

### Costing

Costs per patient per year were estimated by type of hospitals and diagnosis, using patient-level data for information on number of admissions and number of bed-days, and hospital financial records for hospital-level information on total budgets. To arrive at costs per patient per year, the number of bed-days for each patient and diagnosis were multiplied by the relevant unit costs per bed-day, depending on hospital type and diagnosis. An overview of hospital funding information and share of funding sources are presented in Table S1 in the supplementary material. More detailed information about the payment methods and costing estimation is also provided in the supplementary material Table S2.

### Statistical analysis

Statistical analyses were performed in R software version 1.2.5033 by RStudio Inc., Boston US, while data preprocessing of hospital accounting information was performed in MS Excel. More detailed information related to the statistical analysis is reported in the supplementary material. Costs were estimated in Mongolian National Tugrik (2472 MNT = 1 USD in 2018) in real terms at base year in 2018. Findings are reported in international dollars (int$) by using purchasing power parity (PPP$) exchange rates for the period 2016 to 2018 [30].

## Results

**Fig. 3:**
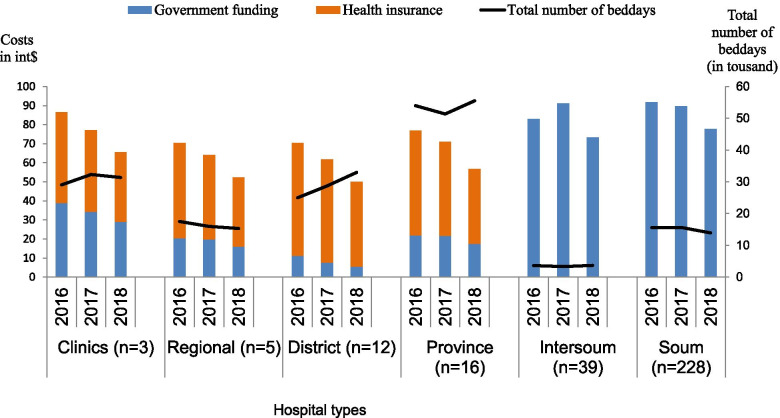
Cost per bed-day by types of hospital and years for stroke, in int$ 2016–2018

Table S3 in the supplementary material summarizes the trends in inpatient care services according to the hospital levels and hospital types. A total of 136.9 thousand admissions were recorded with similar numbers in all years. Almost one third of the inpatient care was delivered by the secondary level provincial hospitals, followed by the primary level community hospitals in rural areas. The largest number of admissions was recorded for patients diagnosed with IHD, followed by admissions for stroke and COPD. Table S4 in the supplementary material presents mean costs per bed-day across different hospital types. The cost for IHD was the highest at int$ 80 per day, followed by COPD (int$ 74) and stroke (int$ 67). Figure 3 illustrates the mean per bed-day costs by types of hospital and years for stroke patients, and the stroke-related total number of bed-days (Fig. 3). Cost per bed-day decreased over time after converting the real price to the 2018 price level and international dollars. When comparing all hospitals, the mean cost per bed-day in the primary level hospitals was the highest, while the total number of bed-days was the least in these hospitals. Similar information for COPD and IHD can be found in the supplementary material Fig. S2-S3.

A total of 117.6 thousand unique patients received inpatient health care. (Supplementary Table S5) The majority of the patients were female and more than half of the patients were pensioners who had at least one comorbidity. In general, male patients tended to stay in hospital longer than female patients. Patients who resided in rural areas more frequently received inpatient care due to stroke and IHD, whereas patients in urban areas were most frequently hospitalized for COPD.

In total, 14 possible patient referral pathways by diagnosis were found and their distribution varied across the diagnoses. (Supplementary Table S6) About 7–22% of the patients directly received inpatient care at tertiary level hospitals, indeed bypassing primary and secondary hospitals.

Table 1 shows the mean inpatient costs distribution for the whole sample over the one-year period. Mean inpatient costs differed significantly according to age group, location, comorbidity, and social status when stratified by type of referral pathway. About one quarter of the patients followed unofficial referral pathways, meaning direct referral to a higher level of hospital. Overall mean inpatient costs for all three diagnoses was int$ 721 (sd = 416) and the amounts increased from int$ 677 for official referral pathways to int$ 849 for unofficial referral pathways. In general, higher costs were seen for men, older age groups, herders and people living in rural areas.

**Table 1:** Distribution of inpatient costs stratified by type of referral pathway

Variables	Overall		Yes/official		No/unofficial		*P* value
	*N* = 117,623		*N* = 87,260		*N* = 30,363		
	mean	sd	mean	sd	mean	sd	
Mean costs in int$	721	(416)	677	(372)	849	(502)	
Gender							NS
Male	739	(444)	690	(396)	862	(528)	
Female	708	(395)	667	(352)	837	(476)	
Age group							
0–40	668	(370)	622	(306)	820	(489)	0.00
40–50	730	(434)	680	(368)	877	(546)	
50–60	718	(422)	676	(378)	842	(510)	
60–70	723	(424)	679	(384)	836	(495)	
70–80	748	(415)	705	(383)	860	(471)	
80+	744	(418)	699	(384)	876	(480)	
Location							
Rural	787	(435)	751	(386)	889	(532)	0.00
Urban	601	(355)	541	(301)	774	(432)	
Comorbidity							
0	717	(406)	675	(361)	840	(497)	0.04
1–2	725	(425)	679	(379)	855	(506)	
Social status							
Formal sector	711	(389)	667	(329)	836	(504)	0.00
Private sector	688	(393)	648	(338)	861	(535)	
Herders	796	(432)	757	(382)	885	(517)	
Pensioners	727	(419)	684	(384)	842	(484)	
Unemployed	702	(421)	653	(357)	859	(528)	
Others	702	(424)	660	(374)	854	(541)	

*SD* Standard deviation

Table 2 summarizes the results of the multivariate regression analysis for total inpatient costs. Annual mean inpatient costs were equivalent to int$ 642 in the reference group (being female patients aged under 40 years old, living in urban areas, who work in the formal sector and followed an official referral path). Inpatient costs for rural patients increased by 29% compared to urban patients. In addition, costs increased by 23% for patients who did not follow the official care pathway. The extra costs associated with inappropriate referral pathway amount to int$ 172 (Table 1) indicating that indeed considerable costs might be saved implementing strategies to maintain official referral pathways. Inpatient care costs fluctuated over the last 2 years of the follow-up period, reflecting changes in the length of stay over time.

**Table 2:** Multivariate analysis of annual inpatient costs per patient for three DRGs

Variables	Coefficient (exponential)	Clustered SE
Observations	117,623	
Intercept		
int$	642.2	(0.009)
Gender (ref = female)		
Male	1.04***	(0.004)
Age group (ref = 0–40)		
40–50	1.08***	(0.007)
50–60	1.09***	(0.006)
60–70	1.10***	(0.008)
70–80	1.14***	(0.009)
80+	1.15***	(0.011)
Location (ref = urban)		
Rural	1.29***	(0.004)
Social status (ref = Formal sector)		
Private sector	0.98**	(0.009)
Herders	1.01	(0.009)
Pensioners	0.98**	(0.009)
Others	0.98**	(0.008)
Comorbidities (ref = 0)		
1–2	1.01*	(0.004)
Official referral (pathways (ref = Yes)		
No	1.23***	(0.005)
Year (ref = 2016)		
2017	1.19***	(0.004)
2018	0.91***	(0.004)

*p < 0.1; **p < 0.05; ***p < 0.01

Generalized linear model, log-link link with clustered standard errors, price level at 2018

The results for the regressions on disease-specific annual inpatient costs per patient are presented in Table S8-S10 in the supplementary material. Across the three diagnoses, the factors that were consistently associated with higher inpatient costs were location, inappropriate referral pathways, older age, male gender and comorbidity. The effects on the costs of covariates were stronger among COPD patients as compared to stroke and IHD patients.

## Discussion

Mean inpatient costs for patients diagnosed with COPD, IHD or stroke over the period 2016 to 2018 in Mongolia per patient per year was int$ 721 (sd = 416), varying from int$ 677 (sd = 372) for official referral pathways to int$ 849 (sd = 502) for unofficial referral pathways. Covariates significantly associated with high inpatient costs were location, age, gender and comorbidity. Our results show that costs per admission over three successive years went down in all hospital types when converting to the real price level in 2018. Although hospital funding increased in real terms, this growth was lower than the inflation rate, and explains the trends in int$ term. Regarding disease-specific inpatient costs, part of the variation in inpatient costs may be explained by variation in the mean length of stay for all three diagnoses. Having comorbidity was associated with high inpatient costs for COPD patients, and less so for stroke and IHD patients. For all three diseases, the main inpatient cost drivers were location, inappropriate referral pathways, older age, and male gender, while social status had only a small effect on the inpatient costs.

Our study shows that referral pathways significantly affect inpatient costs and hence highlights the need for more efficient inpatient care delivery by stimulating the role of primary and secondary level hospital care, and enhancing official referral pathways. Similar pathways have been observed in other low- and middle-income countries, where unnecessary referrals lead to 1–2 month delayed diagnosis, ineffective treatment, and poor provider-patient communication for continued care support [31, 32]. In countries with a large surface area like Mongolia, official referral systems will also reduce travel costs and productivity losses for patients and their families [13].

Comparing the costs currently estimated with previous findings in the literature was challenging due to the different perspectives used (societal or health care system), different cost components included (direct or indirect) and different phases of disease conditions studied. Keeping this heterogeneity of study designs in mind, internationally, a substantial variation in the mean inpatient costs was observed. For stroke patients, the mean annual inpatient costs per patient in China was int$ 5264 for 27 bed-days in 2013, including out-of-pocket payment (24.2%) [23]. Another study conducted among patients hospitalized with acute coronary syndrome in eight Asian countries in 2013 found a mean length of stay of 10 days and per day cost in a critical unit that ranged from int $3210 in Singapore, int$ 284 in asia to int$ 98 in Vietnam [6]. For COPD patients, mean hospitalization costs were US$ 3670 in China [26] and US$ 795 in Vietnam [27]. Our findings are in the low range of these numbers with mean costs of INT $722 (sd = 418), and cost per day below $100. This is in line with per capita health spending from domestic resources which was US$ 92 in Mongolia, but US$ 63 in Vietnam, US$ 250 in China, and US$ 3500 in Japan [33]. Mean length of stay per patient was less than 10 days in our sample compared to a much longer mean length of stay in China (27 days) and Japan (30 days) for patient with stroke. In Mongolia, hospitals tend to assume that DRGs are providing funds for a standard 14 days of treatment. Therefore, current hospital payment mechanisms could be creating a shorter length of stay.

Our finding that age is positively associated with high inpatient costs is consistent with a study in eight Asian countries, [6] whereas a Chinese study found that younger stroke patients had higher inpatient costs than older patients due to better prevention programs [23]. Males had higher inpatient costs and longer length of stay compared to females, confirming findings in previous studies. Almost half of all men smoke and three-quarters consume alcohol compared to ten times fewer women smoking and two times fewer drinking alcohol [34]. Hence, the gender gap in health care use may largely be explained by exposure to modifiable risk factors such as tobacco us [35]. Referral to tertiary level hospitals is associated with significantly higher inpatient costs, supporting the findings from a Chinese study that showed mean inpatient costs in tertiary level hospitals were almost twice the amount of those in primary and secondary hospitals [22].

In rural areas, a total of 277 primary level hospitals were included in our study. Each have approximately 5–15 beds, realizing a total of 150 to 1300 admissions annually. In contrast, tertiary level general hospitals (*n* = 3) in the capital city, deliver 9500 to 25,000 specialized admissions annually. Ensuring equitable and accessible quality health care for all is the main challenge in the Mongolian health system’s efforts to attain UHC, given the extremely low population density in a large part of its territory. Any unofficial policy decision and improper financial incentive can increase overall health expenditure and inefficient use of available resources in health, but also causes serious financial burdens on patients and families. Recent study demonstrated that patients admitted in public hospitals in Mongolia were more likely to suffer from catastrophic health expenditure, due to high out-of-pocket payments and pushing them into poverty [36].

Strength of this study were that we analyzed the potential cost drivers for inpatient care use, investigating the role of patient characteristics and different treatment pathways. Findings represent 3 major NCDs and are based on patient-level data for a large number of hospitals and 3 years.

There were some limitations in this study. As with any analysis based on administrative data, our study lacked clinical information regarding the severity of disease. Therefore, we could not examine costs by severity level. Unit costs were estimated from public funding information using the payment methods applied for different health care providers, rather than activity-based expenditures information. Given that information from 303 hospitals had to be processed, that would have been an insurmountable task with little added value in terms of better estimates of per patient costs. We analyzed costs per patient per year funded by public resources rather than total inpatient costs due to the lack of financial information on out-of-pocket payment and health care use in private clinics. In general out-of-pocket payment covers about 32% of Mongolian healthcare spending in 2018 [33].

Health care costs have increased over the years in response to demographic and epidemiological transitions under way in Mongolia (and other countries in Asia). To progress towards UHC, efficient use of available resources is crucial to controlling constantly increasing costs and growing demand. Previous local evidence found that public spending on NCDs in Mongolia was similar to NCDs spending observed in high-income countries and spending is dominated by inpatient care instead of preventive care [15]. This apparently suggests inefficiency in resource use. To support future policy development, our findings add more detailed patient-level data while revealing that inpatient costs associated with unofficial referral pathways were significantly higher. Policy reform to address the latter would free resources that could be used toward achieving universal health coverage. Various policy efforts can ensure more efficient referral pathways through providing alternative care for patients, altering incentives to care providers, and raising knowledge about efficient admission practice. Other policy options are better coordination and control and official NCD disease management. In sum, it would appear that there is ample room for eliminating unnecessary spending on hospital care. The fact that some of the direct referrals might have been appropriate from a clinical point of view cannot be ruled out. We feel, however, this might be the case in a minor proportion only.

Also, Mongolia has massive opportunities to benefit from proactive prevention and health promoting activities. According to the national survey, one in every three Mongolians has three or more common modifiable risks factors for developing NCDs [34]. In absence of strategic prevention programs, the costs associated with preventable NCDs will continue to grow, resulting in an increased demand for inpatient care. This on top of the demographic trend of an ageing population may result in a non-sustainable financial burden to the patient, health system and society [37].

The current study supports policy makers that have to find ways to deal with this rising financial burden in two ways. First, the regression analyses support further action to reduce unofficial referral pathways, by showing that the latter are more expensive than official referral pathways. A caveat here is that a systematic difference might exist between patients using official and unofficial pathways. However, effective measures should be addressed to control escalation of costs associated with inpatient care in Mongolia.

Second, the estimated unit costs and costs per disease per year could be applied in cost-effectiveness studies to support prevention policy to reduce the incidence of NCDs or their complications. In that case, it should be kept in mind that our estimates are a lower boundary for actual costs, since out-of-pocket costs and costs made in private hospitals were not included.

Our findings thus may inform policy makers on the publicly funded costs for three major NCDs, and could provide useful information to inform future cost-effectiveness studies of disease prevention and health promotion programs related to the three major NCDs in Mongolia and comparable LMICs.

## Conclusion

The inpatient costs for major NCDs were substantially lower when an official referral pathway was followed and a substantial proportion of the inpatient care use consisted of pathways that may indicate unofficial self-referrals in Mongolia. Reducing the unofficial use of inpatient care can improve the resource allocation in health care and prevent further escalation of inpatient costs in the future.

Additionally, this study supports further research into the cost-effectiveness of prevention policies aiming at reducing the burden of NCDs in Mongolia as well as other developing countries.

## Supplementary Information

**Additional file 1: Supplementary file**. **Figure S2** Cost per bed-day by types of hospital and years for COPD, in int$ 2016–2018. **Figure S3** Cost per bed-day by types of hospital and years for IHD, in int$ 2016–2018. **Table S1** Overview of hospital funding mechanisms and share of funding sources. **Table S2** Cost weight used to calculate case-based inpatient costs by DRGs. **Table S3** Distribution of admissions according to the hospital types and according to diagnosis by year. **Table S4** Mean costs per bed-day across different hospital types, in INT$ 2016–2018. **Table S5** Characteristics of patients by type of diagnosis, inpatient data 2016–2018. **Table S6** Patient referral pathways by diagnosis and year. **Table S7** Patient characteristics associated with inpatient costs by age groups, (int$) in 2018. **Table S8** Generalized liner model for COPD patients. **Table S9** Generalized linear model for stroke patients. **Table S10** Generalized linear model for IHD patients.

## Data Availability

The datasets generated during and/or analysed during the current study are available from the corresponding author on reasonable request.

## Abbreviations

COPD: Chronic obstructive pulmonary disease
DRG: Diagnosis-related groups
GLM: Generalized linear model
ICD: International classification of diseases
IHD: Ischemic heart disease
LMICs: Low- and middle-income countries
MNT: Mongolian National Tugrik
NCD: Non-communicable disease
PPPY: Per patient per year
UHC: Universal health coverage
