# Supplementary material for: The costs of inappropriate referral pathways in inpatient care for three major noncommunicable diseases in Mongolia: a national registry-based analysis
Source: BMC Health Serv Res. 2021 Nov 27;21:1280. doi: 10.1186/s12913-021-07281-8 (PMC8626993; doi:10.1186/s12913-021-07281-8)
Supplement: Supplementary file 1 — Additional file 1: Supplementary file. Figure S2 Cost per bed-day by types of hospital and years for COPD, in int$ 2016–2018. Figure S3 Cost per bed-day by types of hospital and years for IHD, in int$ 2016–2018. Table S1 Overview of hospital funding mechanisms and share of funding sources. Table S2 Cost weight used to calculate case-based inpatient costs by DRGs. Table S3 Distribution of admissions according to the hospital types and according to diagnosis by year. Table S4 Mean costs per bed-day across different hospital types, in INT$ 2016–2018. Table S5 Characteristics of patients by type of diagnosis, inpatient data 2016–2018. Table S6 Patient referral pathways by diagnosis and year. Table S7 Patient characteristics associated with inpatient costs by age groups, (int$) in 2018. Table S8 Generalized liner model for COPD patients. Table S9 Generalized linear model for stroke patients. Table S10 Generalized linear model for IHD patients. [file 12913_2021_7281_MOESM1_ESM.docx]

**Article title**: The costs of inappropriate referral pathways in inpatient care for three major noncommunicable diseases in Mongolia: A national registry-based analysis

**Journal name**: BMC Health Services Research

# Supplementary material

Contents

[Supplementary material 1](#_Toc58519507)

[**Selection criteria** 2](#_Toc58519508)

[**Payment methods in Mongolia** 2](#_Toc58519509)

[**Cost per bed-day and cost per patient estimates** 3](#_Toc58519510)

[**Statistical analysis** 4](#_Toc58519511)

[**RESULTS** 7](#_Toc58519512)

[Figure S 2 Cost per bed-day by types of hospital and years for COPD, in int$ 2016-2018 9](#_Toc58519514)

[Figure S 3 Cost per bed-day by types of hospital and years for IHD, in int$ 2016-2018. 10](#_Toc58519515)

[Table S 1 Overview of hospital funding mechanisms and share of funding sources 2](#_Toc58519518)

[Table S 2 Cost weight used to calculate case-based inpatient costs by DRGs 6](#_Toc58519519)

[Table S 3 Distribution of admissions according to the hospital types and according to diagnosis by year. 7](#_Toc58519520)

[Table S 4 Mean costs per bed-day across different hospital types, in INT$ 2016-2018 8](#_Toc58519521)

[Table S 5 Characteristics of patients by type of diagnosis, inpatient data 2016-2018 11](#_Toc58519522)

[Table S 6 Patient referral pathways by diagnosis and year 12](#_Toc58519523)

[Table S 7 Patient characteristics associated with inpatient costs by age groups, (int$) in 2018 13](#_Toc58519524)

[Table S 8 Generalized liner model for COPD patients 14](#_Toc58519525)

[Table S 9 Generalized linear model for stroke patients 15](#_Toc58519526)

[Table S 10 Generalized linear model for IHD patients 16](#_Toc58519527)

# **Selection criteria**

# **Payment methods in Mongolia**

Primary level hospitals are funded by government budget funding only, and such primary health care services are free to citizens. Secondary and tertiary level hospitals are funded by varying combinations of government budget funding (12%-95%), health insurance funding (0-80%) and private funding (<10%). Social health insurance was introduced in 1994 and case-based inpatient care is reimbursed by a fixed base rate for a set of 117 diagnosis-related groups (DRG). For the hospitals this means a flat rate is assumed to capture some variation in cost per case.

**Public funding**: Government budget funding is paid to each hospital based on line-items. A total of 38 line-items are used in public hospitals and its percentage in total hospital revenue ranged between 12% for some tertiary hospitals, 80% for other specialized centers and about 50% of secondary level district/province (aimag) level hospitals. Line-items cover non-admission related costs, such as electricity, buildings and other fixed costs in the hospitals.

**Health insurance** allocates revenue to each contracted public and private hospital using diagnosis related groups of diseases (DRGs). DRGs are clinically similar groups of patients that have the same diagnosis and similar pattern of resource consumption. The share of health insurance in each hospital varies from less than 10% of revenue to more than 80% of hospital revenue. This funding is intended to cover variable costs including medication, test reagents and disposal supplies. A payment rate of 300,000 MNT has been fixed for public hospitals and 175,000 MNT for private hospitals. For each DRG, a case-based admission weight allows to specify this further to the level of cost required for that DRG. DRG weights are presented in Table S1 in this supplementary.

**OOP**: During hospitalization, secondary and tertiary public hospitals charge copayments from the patients which range between 10% at secondary hospitals to 15% at tertiary hospitals.

Table S 1 Overview of hospital funding mechanisms and share of funding sources

| Funding sources | | Number of hospitals | Public funding | Health insurance | Other sources |
| --- | --- | --- | --- | --- | --- |
| Payment mechanism | |  | Line item budget | Case-based DRG funding | Copayment |
| Cost components | |  | Fixed costs: salary, building, equipment, heating, electricity | Variable costs: medication, test reagents, disposal suppliers | |
| Tertiary | State general hospitals | 3 | 12-83% | 7-83% | 4-15% |
| Secondary | Province hospitals | 16 | 58-60% | 30-40% | 1-10% |
|  | District hospitals | 12 |  |  |  |
|  | Regional diagnostic centers | 5 | 60% | 34% | 6% |
| Primary | Inter-soum hospitals | 39 | 75-96% | 4-20% | 0-5% |
|  | Soum hospitals | 228 | 95% |  | 5% |

# **Cost per bed-day and cost per patient estimates**

First, the total number of admissions was quantified for each hospital in a given year. Second, accounting for hospital payment methods, costs per admission were estimated based on government budget funding and social health insurance funding. Third, combined with the information on total number of bed-days by hospital and diagnosis, average costs per bed-day for each diagnosis were estimated for each hospital type as a unit price that could be linked to patient-level data. Finally, costs per person per calendar year (PPPY) were estimated from these patient-level data. The PPPY were used for further statistical analysis and obtained for all diagnoses together as well as for the three main diagnoses separately (COPD, IHD, and stroke). Line-item payment methods were used to transfer government budget funding and case-based diagnosis related group (DRG) payment methods were used to transfer health insurance funds.

Splitting of total costs was performed, to single out costs related to inpatient admissions only. These were then divided by the total number of admissions to find costs per admission by diagnosis in each hospital type.

That is, costs per admission funded by government budget funding in year y, in hospital j were calculated as:

${Costs per admission from budget funding}_{yj}=\left( \frac{{Budget funding source}_{yj}}{{Total number of admissions}_{yj}}*F_{j} \right)$

where year *y* hospital *j*, and F_j_ is the percentage of public funding sources that are allocated to inpatient care units. The value of F_j_ was set at 50% in primary and secondary level hospitals, and 70% in tertiary clinics.

The costs per admission reimbursed from health insurance in year i hospital j for disease k was calculated as:

${Costs per admission from health insurance funding}_{yjk}$= $BR_{y}*{weight}_{k}$

where BR denotes the base rate (fixed base rate of 300 thousand MNT between 2016 and 2018), while weight_k_ denotes the cost weight for diagnosis k. Cost weights can be found in Supplementary material Table 2.

The costs per bed-day were calculated as the sum of cost per admission from budget funding and cost per admission from health insurance funding divided by the total number of bed-days per year, per hospital and per diagnosis:

$${CPBD}_{yjk}= \frac{\sum{(BF}_{yj} +{HIF}_{yjk})*{admissions}_{yjk}}{{Total number of beddays}_{yjk}}$$

Where (*y*) represent the year, (j) indicates the hospital, (k) indicate the diagnosis.

Costs per patient per year (PPY) were calculated for each patient, adding over all three diagnoses and all admissions as well as all hospitals in each calendar year separately.

$${Cost PPY}_{iy}=\sum\sum{\sum CPBD}_{yjk}*{LOS}_{iyjk}$$

Where cost per bed-day in the year y, in hospital j, for the diagnosis k was multiplied with the length of stay for each admission of the patient (i) in the year y, the hospital j, the diagnosis k and patient (i)

Similarly, diagnosis-specific costs PPY were obtained by adding only over admissions related to a specific diagnosis.

$${Cost PPY}_{iyk}=\sum\sum{CPBD}_{yjk}*{LOS}_{iyj}$$

# **Statistical analysis**

Descriptive analyses of demographic variables, distribution of patients over the three diagnoses, as well as the hospital types were performed. Mean costs per bed-day by year and type of hospital were estimated for each diagnosis. Continuous variables were presented as values (mean ±standard deviation), and categorical variables were expressed as percentages.

Analysis of variance (ANOVA) and *X*^2^ tests were used for univariate analyses of continuous and categorical variables, respectively. Costs were presented as arithmetic means with standard deviation (SD), but medians were also presented to investigate skewness. Variable-distributions were tested for (non-)normality using QQ plots.

Multivariate regression analysis was used to investigate the relation between inpatient costs per patient per year and patient characteristics and patient referral pathways.

PPY Inpatient costs = f (gender, age, location of patient, social status, comorbidity index, treatment pathway, year)

Similar models were tested for diagnosis specific costs. A generalized linear model (GLM) with log-link function and a gamma distribution was applied to account for the skewed distributions of costs. Estimated coefficients were reported as the exponential form of a ratio of the mean costs for those in the reference group compared to those in the other categories in a categorical variable and per unit of increase in a continuous variable. Clustered standard errors were estimated at patient-level to take into account repeated records from different years.

Table S 2 Cost weight used to calculate case-based inpatient costs by DRGs

| # | Disease | DRG code | DRG coefficient | Costs paid from health insurance fund to the public hospital,  in MNT, 2018 |
| --- | --- | --- | --- | --- |
| 1 | COPD | J40-J44 | J40-J42=0.8219  J43-J44=0.9903 | 246,570  297,090 |
| 2 | IHD | I20-I25 | I20-I22=0.9706  I23=0.8712 | 291,180  261,360 |
| 3 | Stroke | I60-I69 | I64-I69=0.9309  I53-I63=0.9103 | 279,270  273,090 |

# **RESULTS**

Table S3: Distribution of admissions according to the hospital types and according to diagnosis by year.

| Variables | |  | 2016 | 2017 | 2018 |
| --- | --- | --- | --- | --- | --- |
|  |  |  | N=45,395 | N=45,574 | N=45,963 |
| Hospital levels | Primary | Community hospital | 23.5% | 23.3% | 22.2% |
|  |  | Inter-community | 4.9% | 4.7% | 4.8% |
|  | Secondary | Regional hospital | 9.0% | 8.1% | 7.7% |
|  |  | Province hospital | 32.6% | 31.7% | 32.7% |
|  |  | District hospital | 15.7% | 16.5% | 17.0% |
|  | Tertiary | General hospital | 14.4% | 15.7% | 15.7% |
|  |  |  |  |  |  |
| Diagnosis | COPD | (J40-J44) | 17.6% | 17.7% | 17.5% |
|  | Stroke | (I60-I69) | 36.9% | 37.9% | 39.9% |
|  | IHD | (I20-I25) | 45.5% | 44.4% | 42.6% |

Table S 4 Mean costs per bed-day across different hospital types, in INT$ 2016-2018

| Hospital types | Cost per bed-day reimbursed by health insurance | SD | Cost per bed-day funded by government budget | SD | Mean cost per bed-day | SD |
| --- | --- | --- | --- | --- | --- | --- |
| **COPD** | 47.77 | 6.68 | 42.23 | 21.49 | 74.08 | 25.94 |
| State general hospital | 47.01 | 8.40 | 37.36 | 11.21 | 84.37 | 19.62 |
| Regional hospital | 44.76 | 7.17 | 20.98 | 4.77 | 65.74 | 11.94 |
| Province hospital | 47.54 | 5.61 | 15.41 | 12.66 | 62.96 | 18.26 |
| Distric hospital/clinic | 51.77 | 5.55 | 10.02 | 6.66 | 61.80 | 12.22 |
| Inter_soums | 0.00 | 0.00 | 82.94 | 52.55 | 82.94 | 52.55 |
| Soum hospital | 0.00 | 0.00 | 86.67 | 41.06 | 86.67 | 41.06 |
| **IHD** | 51.31 | 8.53 | 45.74 | 19.38 | 79.94 | 25.07 |
| State general hospital | 53.68 | 12.72 | 44.87 | 10.99 | 98.55 | 23.71 |
| Regional hospital | 47.62 | 7.62 | 22.34 | 4.62 | 69.97 | 12.24 |
| Province hospital | 49.92 | 8.45 | 17.42 | 12.30 | 67.34 | 20.74 |
| Distric hospital/clinic | 54.02 | 5.32 | 9.18 | 5.71 | 63.20 | 11.03 |
| Inter_soums | 0.00 | 0.00 | 90.93 | 39.88 | 90.93 | 39.88 |
| Soum hospital | 0.00 | 0.00 | 89.67 | 42.81 | 89.67 | 42.81 |
| **Stroke** | 46.58 | 14.24 | 20.29 | 12.18 | 66.87 | 26.41 |
| State general hospital | 42.30 | 10.55 | 34.07 | 10.70 | 76.37 | 21.25 |
| Regional hospital | 43.57 | 19.51 | 18.69 | 10.86 | 62.25 | 30.37 |
| Province hospital | 47.80 | 14.78 | 20.35 | 14.71 | 68.16 | 29.49 |
| Distric hospital/clinic | 52.64 | 12.10 | 8.06 | 12.43 | 60.69 | 24.53 |
| Inter_soums | 0.00 | 0.00 | 82.47 | 63.91 | 82.47 | 63.91 |
| Soum hospital | 0.00 | 0.00 | 86.41 | 60.60 | 86.41 | 60.60 |

COPD, Chronic obstructive pulmonary disease; IHD, Ischemic heart disease; SD, standard deviation;

Figure S 1 Cost per bed-day by types of hospital and years for COPD, in int$ 2016-2018

Figure S 2 Cost per bed-day by types of hospital and years for IHD, in int$ 2016-2018.

Table S 5 Characteristics of patients by type of diagnosis, inpatient data 2016-2018

| **Variables** | **Patients diagnosed with COPD** | | | **Patients diagnosed with stroke** | | | **Patients diagnosed with IHD** | | |
| --- | --- | --- | --- | --- | --- | --- | --- | --- | --- |
| Year | 2016 | 2017 | 2018 | 2016 | 207 | 2018 | 2016 | 207 | 2018 |
| Number of patients | **7,442.00** | **7,234.00** | **7,067.00** | **14,352.00** | **13,758.00** | **14,696.00** | **19,047.00** | **16,781.00** | **17,257.00** |
| Male | 43.8% | 46.9% | 47.2% | 44.7% | 43.5% | 42.5% | 44.1% | 45.3% | 44.4% |
| Female | 56.2% | 53.1% | 52.8% | 55.3% | 56.5% | 57.5% | 55.9% | 54.7% | 55.6% |
| Mean age (sd) |  |  |  |  |  |  |  |  |  |
| Male | 57.3(16.8) | 58.9(16.1) | 58.1(16.4) | 56.2(14.3) | 57.3(13.7) | 57.3(13.7) | 59(13.9) | 59.2(13.8) | 59(13.9) |
| Female | 54.4(17.9) | 57.5(16.8) | 55.48(17.7) | 57.3(14.2) | 58.5(14.2) | 59(13.9) | 60.3(14.7) | 60.6(14.9) | 60.7(14.8) |
| Location |  |  |  |  |  |  |  |  |  |
| Rural | 52.5% | 62.1% | 54.7% | 60.8% | 67.0% | 58.4% | 69.5% | 74.0% | 69.5% |
| Urban | 47.5% | 37.9% | 45.3% | 39.2% | 33.0% | 41.6% | 30.5% | 26.0% | 30.5% |
| Social status |  |  |  |  |  |  |  |  |  |
| Herders | 6.0% | 7.4% | 6.6% | 5.4% | 6.8% | 5.7% | 8.9% | 9.1% | 8.9% |
| Pensioners | 48.2% | 52.1% | 49.0% | 49.5% | 51.1% | 52.6% | 55.9% | 55.3% | 54.7% |
| Formal sector | 8.8% | 7.3% | 7.9% | 6.0% | 6.1% | 6.4% | 6.8% | 6.4% | 6.9% |
| Private business | 9.2% | 8.0% | 9.1% | 5.7% | 6.2% | 7.0% | 6.8% | 6.9% | 8.1% |
| Others | 27.9% | 25.3% | 27.4% | 33.3% | 29.8% | 28.3% | 21.5% | 22.3% | 21.4% |
| Mean length of stay (sd) | |  |  |  |  |  |  |  |  |
| Male | 9.26 (5.64) | 9.26 (4.58) | 9.7(6.31) | 10.3(5.66) | 10.9(6.35) | 10.9(6.57) | 8.45(2.97) | 9.24(4.01) | 8.5(3.58) |
| Female | 8.51 (3.15) | 8.66 (3.4) | 8.38(3.56) | 9.92(5.19) | 10.6(5.89) | 10(5.84) | 8.77(4.08) | 9.43(4.44) | 8.97(4.73) |
| Comorbidity index | |  |  |  |  |  |  |  |  |
| 0 | 40% | 32% | 36% | 41% | 33% | 37% | 48% | 36% | 43% |
| 1-2 | 60% | 68% | 64% | 58% | 67% | 63% | 52% | 64% | 57% |

Table S 6 Patient referral pathways by diagnosis and year

| **Variables** | **Patients diagnosed with COPD** | | | **Patients diagnosed with stroke** | | | **Patients diagnosed with IHD** | | |
| --- | --- | --- | --- | --- | --- | --- | --- | --- | --- |
| Year | 2016 | 2017 | 2018 | 2016 | 2017 | 2018 | 2016 | 2017 | 2018 |
| Number of patients | **7,442** | **7,234.** | **7,067** | **14,352** | **13,758** | **14,696** | **19,047** | **16,781** | **17,257** |
| Referral pathways^[[1]](#footnote-1)^ |  |  |  |  |  |  |  |  |  |
| 1-0-0 | 22.6% | 21.4% | 24.0% | 14.2% | 20.4% | 12.6% | 41.5% | 35.9% | 41.0% |
| 1-2-0 | 0.3% | 1.1% | 0.4% | 1.3% | 1.8% | 1.2% | 0.6% | 3.8% | 0.6% |
| 1-3-0 | 0.1% | 0.6% | 0.1% | 0.4% | 0.6% | 0.6% | 0.1% | 0.6% | 0.2% |
| 1-2-3 | 0.03% | 0.01% | 0.03% | 0.1% | 0.2% | 0.2% | 0.0% | 0.0% | 0.0% |
| 1-3-2 | 0.0% | 0.0% | 0.0% | 0.1% | 0.1% | 0.1% | 0.0% | 0.0% | 0.0% |
| 2-0-0 | 53.9% | 52.5% | 52.4% | 65.1% | 57.0% | 64.8% | 48.9% | 46.1% | 47.8% |
| 2-1-0 | 0.2% | 0.5% | 0.3% | 0.3% | 1.2% | 0.5% | 0.3% | 2.2% | 0.4% |
| 2-3-0 | 0.8% | 1.2% | 1.1% | 2.3% | 2.9% | 3.2% | 0.7% | 1.3% | 0.9% |
| 2-3-1 | 0.0% | 0.0% | 0.0% | 0.0% | 0.0% | 0.1% | 0.0% | 0.0% | 0.0% |
| 2-1-3 | 0.0% | 0.0% | 0.0% | 0.0% | 0.1% | 0.0% | 0.0% | 0.0% | 0.0% |
| 3-0-0 | 21.6% | 21.7% | 20.6% | 14.5% | 13.3% | 13.8% | 7.5% | 9.2% | 8.5% |
| 3-2-0 | 0.3% | 0.8% | 0.8% | 1.5% | 2.1% | 2.6% | 0.3% | 0.5% | 0.5% |
| 3-1-0 | 0.1% | 0.2% | 0.1% | 0.1% | 0.2% | 0.2% | 0.0% | 0.3% | 0.1% |
| 3-1-2 | 0.0% | 0.01% | 0.03% | 0.0% | 0.0% | 0.0% | 0.01% | 0.01% | 0.02% |

Table S 7 Patient characteristics associated with inpatient costs by age groups, (int$) in 2018

| Variables | 0-39 | | 40-64 | | 65+ | | P value |
| --- | --- | --- | --- | --- | --- | --- | --- |
|  | N=13027 | | N=64133 | | N=40463 | |  |
|  | mean | (sd) | mean | (sd) | mean | (sd) |  |
| Gender |  |  |  |  |  |  |  |
| Male | 673 | (376) | 738 | (452) | 762 | (450) | 0.008** |
| Female | 661 | (355) | 707 | (396) | 723 | (397) |  |
| Location |  |  |  |  |  |  |  |
| Rural | 723 | (376) | 788 | (438) | 807 | (440) | 0.003** |
| Urban | 546 | (305) | 592 | (359) | 628 | (358) |  |
| Comorbidity |  |  |  |  |  |  |  |
| 0 | 670 | (363) | 715 | (415) | 732 | (403) | 0.002** |
| 1-2 | 664 | (365) | 725 | (428) | 745 | (432) |  |
| Referral paths |  |  |  |  |  |  |  |
| Yes | 622 | (306) | 677 | (375) | 696 | (387) | 0.000*** |
| No | 820 | (489) | 852 | (519) | 853 | (480) |  |
| Social status |  |  |  |  |  |  |  |
| Formal sector | 674 | (358) | 726 | (399) | 687 | (407) | 0.172 |
| Private sector | 623 | (335) | 709 | (407) | 744 | (464) |  |
| Herders | 741 | (371) | 812 | (449) | 822 | (414) |  |
| Pensioners | 688 | (609) | 707 | (416) | 739 | (420) |  |
| Unemployed | 651 | (379) | 715 | (421) | 760 | (364) |  |
| Others | 674 | (382) | 712 | (437) | 745 | (412) |  |

P-values are based on the Anova test. SD Standard deviation

Table S 8 Generalized liner model for COPD patients

| COPD | Basic model | | Extended model | |
| --- | --- | --- | --- | --- |
|  | Coefficient (Exponential) | Clustered SE | Coefficient (Exponential) | Clustered SE |
| Observation N |  |  | 21,739 |  |
| Intercept |  |  |  |  |
| int$ | 474.71*** | (0.01) | 480.07*** | (0.014) |
| Sex (ref=female) |  |  |  |  |
| Male | 1.07*** | (0.007) | 1.07*** | (0.008) |
| Age group (ref= < 40) |  |  |  |  |
| 40-50 | 1.10*** | (0.012) | 1.14*** | (0.013) |
| 50-60 | 1.10*** | (0.01) | 1.13*** | (0.011) |
| 60-70 | 1.12*** | (0.014) | 1.17*** | (0.016) |
| 70-80 | 1.14*** | (0.015) | 1.22*** | (0.017) |
| 80+ | 1.14*** | (0.017) | 1.22*** | (0.02) |
| Location (ref=Urban) |  |  |  |  |
| Rural | 1.13*** | (0.007) | 1.27** | (0.008) |
| Social status (ref=Formal sector) | |  |  |  |
| Private sector | 0.96* | (0.013) | 0.95** | (0.015) |
| Herders | 1.00 | (0.016) | 1.01 | (0.017) |
| Pensioners | 1.00 | (0.015) | 1.02 | (0.016) |
| Others | 0.98 | (0.014) | 0.99 | (0.015) |
| Official referral (ref=Yes) | |  |  |  |
| No | 1.15*** | (0.007) | 1.19*** | (0.008) |
| Comorbidity index (ref=0) |  |  |  |  |
| 1-2 |  |  | 1.00 | (0.007) |
| Year (ref=2016) |  |  |  |  |
| 2017 |  |  | 1.21*** | (0.008) |
| 2018 |  |  | 0.90*** | (0.009) |

Clustered standard errors at individual level in parentheses;

*p<0.1; **p<0.05; ***p<0.01

Table S 9 Generalized linear model for stroke patients

| Stroke |  | Basic model | | Extended model | |
| --- | --- | --- | --- | --- | --- |
|  |  | Coefficient (Exponential) | Clustered SE | Coefficient (Exponential) | Clustered SE |
| Observation N |  |  |  | 42802 |  |
| Intercept |  |  |  |  |  |
| int $ |  | 504.82*** | (0.014) | 525.12*** | (0.015) |
| Sex (ref=female) |  |  |  |  |  |
| Male |  | 1.05*** | (0.006) | 1.06*** | (0.006) |
| Age group (ref= < 40) |  |  |  |  |  |
| 40-50 |  | 1.08*** | (0.011) | 1.08*** | (0.011) |
| 50-60 |  | 1.05*** | (0.011) | 1.05*** | (0.011) |
| 60-70 |  | 1.00 | (0.013) | 1.03*** | (0.013) |
| 70-80 |  | 1.01 | (0.014) | 1.06*** | (0.014) |
| 80+ |  | 0.99 | (0.017) | 1.06*** | (0.018) |
| Location (ref=Urban) |  |  |  |  |  |
| Rural |  | 1.20*** | (0.006) | 1.28 | (0.006) |
| Social status (ref=Formal sector) |  |  |  |  |  |
| Private sector |  | 1.04** | (0.016) | 1.03* | (0.016) |
| Herders |  | 1.03** | (0.017) | 1.01 | (0.016) |
| Pensioners |  | 1.05* | (0.014) | 1.04 | (0.014) |
| Others |  | 1.03 | (0.013) | 1.01 | (0.013) |
| Treatment pathway (ref=Yes) |  |  |  |  |  |
| No |  | 1.21*** | (0.006) | 1.25*** | (0.006) |
| Comorbidity index (ref=0) |  |  |  |  |  |
| 1-2 |  |  |  | 1.01 | (0.006) |
| Year (ref=2016) |  |  |  |  |  |
| 2017 |  |  |  | 1.17*** | (0.007) |
| 2018 |  |  |  | 0.91*** | (0.007) |

Clustered standard errors at individual level in parentheses;

*p<0.1; **p<0.05; ***p<0.01

Table S 10 Generalized linear model for IHD patients

| IHD | Basic model | | Extended model | |
| --- | --- | --- | --- | --- |
|  | Coefficient (Exponential) | Clustered SE | Coefficient (Exponential) | Clustered SE |
| Observation N |  |  | 53082.00 |  |
| Intercept |  |  |  |  |
| int$ | 501.19*** | (0.009) | 516.21*** | (0.01) |
| Sex (ref=female) |  |  |  |  |
| Male | 1.02*** | (0.004) | 1.02*** | (0.005) |
| Age group (ref= < 40) |  |  |  |  |
| 40-50 | 1.05*** | (0.007) | 1.05*** | (0.008) |
| 50-60 | 1.06*** | (0.007) | 1.07*** | (0.008) |
| 60-70 | 1.10*** | (0.009) | 1.11*** | (0.01) |
| 70-80 | 1.14*** | (0.01) | 1.15*** | (0.011) |
| 80+ | 1.16*** | (0.011) | 1.16*** | (0.013) |
| Location (ref=Urban) |  |  |  |  |
| Rural | 1.26*** | (0.005) | 1.30*** | (0.005) |
| Social status (ref=Formal sector) |  |  |  |  |
| Private sector | 0.96*** | (0.009) | 0.95*** | (0.01) |
| Herders | 0.94** | (0.009) | 0.95* | (0.01) |
| Pensioners | 0.97*** | (0.01) | 0.98*** | (0.01) |
| Others | 0.95 | (0.01) | 0.95*** | (0.01) |
| Official referral (ref=Yes) |  |  |  |  |
| No | 1.14*** | (0.006) | 1.15*** | (0.006) |
| Comorbidity index (ref=0) |  |  |  |  |
| 1-2 |  |  | 1.01** | (0.004) |
| Year (ref=2016) |  |  |  |  |
| 2017 |  |  | 1.21 | (0.005) |
| 2018 |  |  | 0.90 | (0.005) |

Clustered standard errors at individual level in parentheses;

*p<0.1; **p<0.05; ***p<0.01

1. Patient referral pathways defined based on what referrals took place within a year. For example:

   1-0-0 indicates the patient received inpatient care only at a primary level hospital (1)

   1-2-0 indicates the patient received inpatient care at a primary level hospital (1) and was referred to a secondary level hospital (2)

   1-3-0 indicates the patient received inpatient care first at a primary level hospital and self-transferred to a tertiary level hospital (3).

   1-2-3 indicates the patient received inpatient care at a primary level hospital (1), secondary level (2) and tertiary level hospital (3) [↑](#footnote-ref-1)
